# Prevalence and correlates of fecal incontinence among nursing home residents: a population-based cross-sectional study

**DOI:** 10.1186/1471-2318-13-87

**Published:** 2013-08-30

**Authors:** Susan Saga, Anne Guttormsen Vinsnes, Siv Mørkved, Christine Norton, Arnfinn Seim

**Affiliations:** 1 Faculty of Nursing, Sør-Trøndelag University College, Trondheim, 7004, Norway; 2 Department of Public Health and General Practice, Norwegian University of Science and Technology, Trondheim, 7491, Norway; 3 Clinical Service, St. Olavs Hospital, Trondheim University Hospital, Trondheim, 7006, Norway; 4 Florence Nightingale School of Nursing and Midwifery, King’s College London, Waterloo Road, London, UK

**Keywords:** Fecal incontinence, Nursing homes, Residential facilities, Homes for the aged, Frail elderly, Cross sectional study, Prevalence studies, Epidemiologic study

## Abstract

**Background:**

Fecal incontinence is highly prevalent among nursing home residents. Previous nursing home studies have identified co-morbidity associated with fecal incontinence, but as this population is increasingly old and frail, we wanted to see if the rate of fecal incontinence had increased and to investigate correlates of fecal incontinence further.

**Methods:**

Cross-sectional study of the entire nursing home population in one Norwegian municipality. Registered nurses filled in a questionnaire for all residents in the municipality (980 residents aged ≥65). Statistical methods used are descriptive statistics, binary logistic regression and multivariable logistic regression.

**Results:**

The response rate of the study was 90.3%. The prevalence of fecal incontinence was 42.3%. In multivariable analysis of FI, residents with diarrhea (OR 7.33, CI 4.39-12.24), urinary incontinence (OR 2.77, CI 1.73-4.42) and dementia (OR 2.17, CI 1.28-3.68) had higher odds of having fecal incontinence compared to those without the condition. Residents residing in a nursing home between 4–5 years had higher odds of having fecal incontinence compared to residents who had stayed under a year (OR 2.65, CI 1.20-5.85). Residents with deficiency in feeding (2.17, CI 1.26-3.71), dressing (OR 4.03, CI 1.39-11.65), toilet use (OR 7.37, CI 2.65-20.44) and mobility (OR 2.54, CI 1.07-6.00) had higher odds of having fecal incontinence compared to residents without deficiencies in activities of daily living (ADL). Needing help for transfer between bed and chair was a protective factor for fecal incontinence compared to residents who transferred independently (OR 0.49, CI 0.26-0.91).

**Conclusions:**

Fecal incontinence is a prevalent condition in the nursing home population and is associated with ADL decline, frailty, diarrhea and quality of care. This knowledge is important for staff in nursing home in order to provide the best treatment and care for residents with fecal incontinence.

## Background

The prevalence of fecal incontinence is high among older residents in nursing homes [1]. Fecal incontinence is a bothersome condition and has a significant impact for both resident and caregivers. Residents in nursing homes have lost many functions, but losing control over bodily functions such as emptying the bowels, is most closely related to loss of dignity. This is a great professional challenge for the caregivers involved. Fecal incontinence is the involuntary loss of liquid or solid stool that is a social or hygienic problem [1]. Prevalence of fecal incontinence in community-dwelling people over 60 years is 5.1-6.2% and age has an important influence on the rate of fecal incontinence [2]. In nursing homes, however, previous studies suggest a prevalence between 10.3% [3] and 63.6% [4], but is more often reported to be somewhere between 40 and 55% [5-10]. This difference in prevalence in community-dwelling people over 60 years compared to older nursing home residents is major.

The number of nursing home residents has risen worldwide in recent decades. Medical advances and improvements in public health have led to an increase in life expectancy, however often with accompanying morbidities [11]. Life expectancy will continue to increase in the next 30 years [12]. It is expected that the nursing home population will therefore grow in the future. A nursing home is a place of residence for people with health problems and significant deficiencies in activities of daily living (ADL). In Norway, the municipalities have a statutory obligation to provide nursing home services to those who need it. Consequently, all nursing homes are accounted for and subject to governmental control. Most Norwegian nursing homes are owned and run by the municipality, and financed by taxes and resident payment. However, there are also some private non-profit and for-profit providers [13]. The nursing homes have nurses on duty 24-hours a day and the staff comprises some registered nurses, but mostly licensed practical nurses, while some are unskilled. Most nursing home residents in Norway are long-term care residents, but the number of short-term care residents is increasing. Laws and regulations are providing a framework for how nursing homes in Norway are managed and organized, and secure a relatively homogenous public service across the country. Nursing home residents and nursing home institutions worldwide have gone through a great change during the past decades, with increasingly frail residents with multiple comorbidities. However, many fecal incontinence studies are from the 80s and 90s [4-7,9,10,14-16], but there are some studies from the past decade [3,8,17,18]. Previous studies have demonstrated the complexity of fecal incontinence among frail older residents. In this group, fecal incontinence may be an indicator of generalized bowel dysfunction rather than an isolated anal sphincter problem as it may be in a younger population [19]. Bowel-symptoms such as constipation and diarrhea are related to fecal incontinence [7,20,21] as well as laxative use [6], but in this group there is also a strong relationship to reduced mobility [3,5,7,18,20,21], decline in cognitive function [5-7,9,20,21], in addition to diseases like diabetes [3,22], cerebral stroke [3,6,10], Parkinson’s disease [23], depression [24] and urinary incontinence [3,7,10].

The aim for fecal incontinence care and treatment is to achieve reduction in bowel leakage and accidents for residents. However, this is a group of frail elderly residents with the risk of further deterioration. An older and frailer nursing home population could possibly lead to a higher prevalence of fecal incontinence. It is therefore important to establish knowledge about fecal incontinence in this growing group of vulnerable residents. We wanted to take a detailed look at factors that registered nurses could potentially improve and to provide new insights for registered nurses. Hence, the aim of our study was to assess the prevalence and further explore correlates of fecal incontinence in the nursing home population.

## Methods

This cross-sectional study was performed in nursing homes in Trondheim municipality, Norway during June 2010. The Regional Committee for Medical and Health research Ethics, REC South East, approved the study. No consent from residents or their next of kin was required because all the resident information gathered was de-identified and anonymous when given to the researcher.

### Design and participants

**Figure 1:**
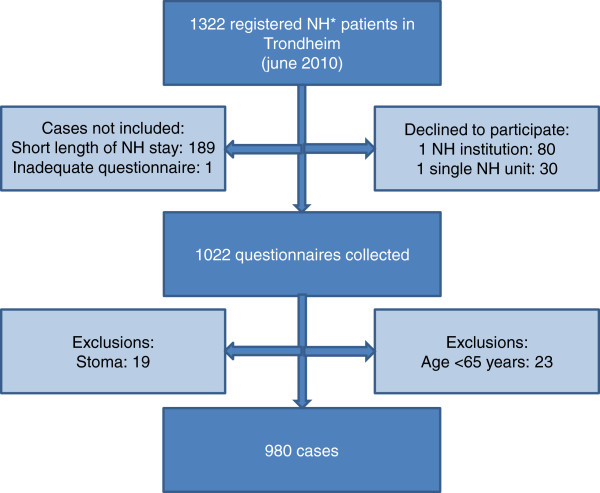
**Flow chart of inclusions and exclusions.** *nursing home.

**Figure 2:**
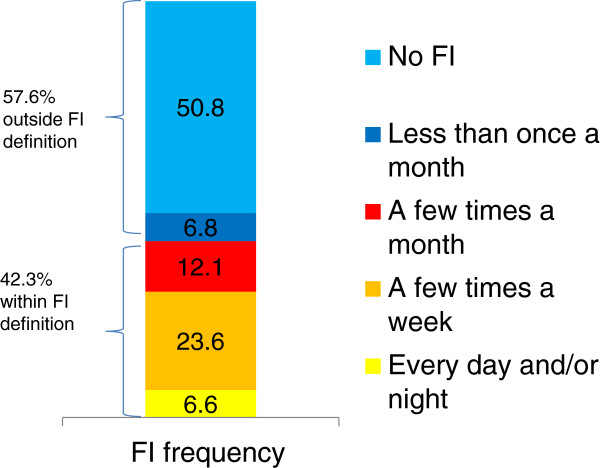
Prevalence and frequency of fecal incontinence.

Trondheim is the third largest municipality in Norway, consisting of both urban and rural areas. There are 28 nursing homes in Trondheim, mostly owned by the municipality; however, there are also a few non-profit private providers. Information regarding all nursing homes was gathered from the municipal administration. All 28 nursing homes in the municipality were invited to participate. One nursing home and a single unit at another nursing home declined. Residents who at the time of data collection were residents in a nursing home were included if they had been a resident for more than three weeks or had prior stays of more than four weeks during the last six months. Residents who were younger than 65 years, as well as residents with a stoma, were excluded from the study after the data collection. Registered nurses filled in a questionnaire for all the residents that met the inclusion criteria in the 27 participating nursing homes. This gives a response rate of 100% for the participating nursing home units and 90.3% response rate for the entire nursing home population in the municipality. The total number of nursing home residents in the municipality was at that point of time 1322, whereas the total number of cases after exclusions was 980 (Figure 1). Registered nurses with comprehensive oversight of each resident filled in the questionnaires. The municipality received payment as a compensation for the time used. Fecal incontinence in this study was defined as involuntary leakage of stool at least a few times a month (Figure 2).

### Measures

A questionnaire was designed for this study in order to obtain information about fecal incontinence, constipation, diarrhea, sex, age, diagnoses, medications, type of care and length of stay. In the development of the questionnaire, a group consisting of nurses, a physiotherapist and a physician participated, all of whom had experience from research, clinical practice and education within the area of incontinence, thereby establishing face/content validity as an expert group. The pilot testing was conducted in one nursing home unit by several registered nurses. A research nurse administered the pilot testing, and gathered feedback regarding the questionnaire from the participating nurses (i.e. understandable questions, ambiguous questions, adequate or lacking answering alternatives). We revised the questionnaire according to the accumulated feedback from the pilot test. In the questionnaire the registered nurses were asked to answer “yes” or “no” to the question: “Does the resident have involuntary leakage of feces?” If “yes”, the registered nurses were asked to indicate whether the resident had had involuntary leakage of feces less than once a month, a few times a month, a few times a week or every day and/or night. The same question and labeling were used to ask about urinary incontinence. Frequency labeling is taken from Sandvik et al. [25]. Both urinary incontinence and fecal incontinence in this study were defined as involuntary leakage of urine/stool at least a few times a month. The registered nurses were also asked about constipation: “According to your assessment, is the resident constipated?” This question had three alternative answers: (1) “Yes”, (2) “no” and (3) “no, because the resident is using laxatives”. In this study we have chosen to include both (1) and (3) in our definition of constipation, i.e. we have included laxative use in our definition. The variable “cognitive impairment” is based on the question: “According to your assessment, is the resident aware of the current time, place and situation?” The registered nurses could answer “yes,” “no” or “partly” to this question. Both “no” and “partly” are taken as cognitive impairment in this paper. The registered nurses were also asked to report all diagnoses and medications residents used regularly from the medical record: Providing the variables diabetes, stroke, Parkinson’s disease, hip problems (hip fractures, current symptoms or problems from the hip and current coxarthritis), depression and sight reduction. To obtain information about the residents’ Activities of Daily Living (ADL)-functioning Barthel’s ADL index was used, with the scores from 0–20 (20 is the best score) [26]. We used both ADL score and separate questions from Barthel’s ADL index in statistical analysis.

### Statistical analysis

Statistical methods included estimating prevalence in percentages, and other descriptive statistics. Binary logistic regression analysis was conducted on 21 variables, identified in previous studies, to measure strength of association and to identify possible risk factors: Age, sex, length of stay in nursing home, constipation, diarrhea, urinary incontinence, dementia, diabetes, stroke, Parkinson’s disease, hip problems, depression, sight reduction and deficiencies in feeding, grooming, dressing, transfer, toilet use, mobility, walking in stairs and bathing. Variables significantly associated with fecal incontinence in bivariate logistic regression analysis were entered as categorical variables in the multivariable logistic regression analysis. Multivariable logistic regression was conducted to measure the strength of associations between the independent variables and the dependent variable (fecal incontinence). Effect sizes are presented as odds ratio (OR) with 95% CI and p-values. Variables were considered significant if p < 0.05, but p-values between 0.01 and 0.05 were interpreted with caution. The Nagelkerke R square test for goodness-of-fit was used to assess how well the chosen model fitted the data. No replacements were made for missing data. Due to some missing data the number of residents varies between the different analyses: N varies between 943 and 980 in bivariate analysis and N = 868 in multivariable logistic regression. Statistical calculations were performed using PASW® statistics 19 for Windows (SPSS Inc., Chicago, Illinois USA).

## Results

Table 1 demonstrates that mean age of the included residents was 85.5 years (SD 7.3), ranging from 65 to 107 years. Women constituted 73.9% and men 26.1% of the nursing home population. Of the residents, 92.5% were in long-term care, whereas the remaining 7.5% were in short-term care, including rehabilitation and respite stays (Table 1). Mean duration of stay among residents in short-term care was 51.1 (SD 56.6) days, whereas mean duration of residency in long-term care was 881.9 (SD 871.0) days. Table 1 demonstrates that cognitive impairment was reported in 80.3% of the residents. Mean score on Barthel’s ADL index was 9.5 (SD 5.6).

**Table 1:** Demographic and medical characteristics of nursing home residents

**Characteristic**	**Value**
Age, mean ± SD	85.5 ± 7.3
Gender	
*Men, n (%)*	253 (26.1)
*Women, n (%)*	717 (73.9)
Length of stay in nursing home, mean ± SD	827.2 ± 882.1
ADL score, mean ± SD	9.5 ± 5.6
Type of stay in nursing home	
*Short-term care, n (%)*	71 (7.5)
*Long-term care, n (%)*	878 (92.5)
Cognitive impairment, n (%)	778 (80.3)

### Prevalence and severity of fecal incontinence

Nurses reported that 42.3% of the residents had involuntary leakage of feces a few times a month or more (Figure 2). The most prevalent group had involuntary leakage of feces a few times a week (23.6% of the nursing home residents). Urgency of defecation was reported in 52.7% of the residents.

### Prevalence of fecal incontinence according to sex, age, type of care and length of stay

There was no significant difference in prevalence of fecal incontinence between men (37.5%) and women (43.9%) (p = 0.08). The different age groups had a prevalence of fecal incontinence ranging from 37.3 to 53.8, but there were no significant differences, trends or patterns (p = 0.68) (Table 2). Among residents in long-term care, 44.1% had fecal incontinence, while residents in short-term care had a prevalence of 17.3% (p < 0.001). However, prevalence of fecal incontinence increased significantly with increasing length of residency up to 5 years (p < 0.001) (Table 2). When adjusted for other factors, only residents that had been in the institution between 4 and 5 years had significantly higher odds of having fecal incontinence compared to those who had stayed in nursing home under a year (Table 3).

**Table 2:** Prevalence of fecal incontinence in percentages by age and length of stay in nursing home

***Variable (n)***	***Prevalence of fecal incontinence (%)***	***P-value ***^***a***^
**Age groups (975)**	-	0.68
*65-69 years (28)*	42.9	
*70-74 years (59)*	37.3	
*75-79 years (104)*	46.2	
*80-84 years (189)*	38.6	
*85-89 years (299)*	45.2	
*90-94 years (214)*	39.3	
*95-99 years (69)*	43.5	
*>100 years (13)*	53.8	
**Length of stay in NH (943)**	-	<0.001
*<1 year (354)*	28.5	
*1-2 years (198)*	42.4	
*2-3 years (125)*	46.4	
*3-4 years (95)*	55.8	
*4-5 years (56)*	69.6	
*>5 years (115)*	55.7	

^a^Chi squared test.

**Table 3:** **Correlates of fecal incontinence in bivariate and multivariable analysis **^**b**^

***Independent variables***	***Variable alternative***	***Unadjusted OR (95% CI)***	***Adjusted OR ***^***c ***^***(95% CI)***	***P-value ***^***d***^
**Length of stay**	*<1 year*	1.00 (Reference)	1.00 (Reference)	-
	*1-2 years*	1.85 (1.28-2.66)	1.36 (0.84-2.20)	0.21
	*2-3 years*	2.17 (1.42-3.30)	1.18(0.67-2.06)	0.57
	*3-4 years*	3.16 (1.98-5.04)	1.46 (0.79-2.69)	0.23
	*4-5 years*	5.75 (3.11-10.63)	2.65 (1.20-5.85)	0.02
	*>5 years*	3.14 (2.04-4.85)	1.51 (0.84-2.71)	0.17
**Constipation**		1.55 (1.20-2.01)	1.04 (0.72-1.50)	0.83
**Diarrhea**		6.20 (4.17-9.23)	7.33 (4.39-12.24)	<0.001
**Urinary incontinence**		6.39 (4.58-8.90)	2.77 (1.73-4.42)	<0.001
**Dementia**		3.85 (2.62-5.67)	2.17 (1.28-3.68)	0.004
**Stroke**		1.67 (1.16-2.40)	1.09 (0.64-1.84)	0.76
**Feeding**	*Independent*	1.00 (Reference)	1.00 (Reference)	-
	*Needs help cutting, spreading butter etc.*	2.70 (1.95-3.73)	1.21 (0.78-1.88)	0.39
	*Unable*	7.31 (5.10-10.48)	2.17 (1.26-3.71)	0.005
**Grooming**	*Independent*	1.00 (Reference)	1.00 (Reference)	
	*Dependent*	0.12 (0.07-0.22)	2.48 (0.87-7.08)	0.09
**Dressing**	*Independent*	1.00 (Reference)	1.00 (Reference)	-
	*Needs help, but can do about half unaided*	4.38 (2.29-8.40)	2.41 (0.92-6.28)	0.07
	*Dependent*	20.86 (11.32-38.45)	4.03 (1.39-11.65)	0.01
**Transfer**	*Independent*	1.00 (Reference)	1.00 (Reference)	-
	*Some help needed*	2.25 (1.54-3.28)	0.49 (0.26-0.91)	0.03
	*Can sit, but needs a lot of help*	6.60 (4.57-9.53)	0.61 (0.28-1.35)	0.22
	*Can’t sit, lift used*	12.14 (7.84-18.81)	0.52 (0.19-1.43)	0.21
**Toilet use**	*Independent*	1.00 (Reference)	1.00 (Reference)	-
	*Help needed for dressing/transfer*	8.20 (5.64-11.92)	3.13 (1.61-6.06)	0.001
	*Can’t use toilet/toilet chair*	41.98 (21.73-81.12)	7.37 (2.65-20.44)	<0.001
**Mobility**	*Walks*	1.00 (Reference)	1.00 (Reference)	0.04
	*Walks with support*	1.79 (1.25-2.56)	1.47 (0.83-2.59)	0.19
	*Can’t walk, but able to move wheelchair*	2.24 (1.25-4.03)	0.90 (0.35-2.35)	0.83
	*Immobile*	10.86 (7.16-16.45)	2.54 (1.07-6.00)	0.03
**Stairs**	*Independent up* &*down*	1.00 (Reference)	1.00 (Reference)	-
	*Needs help from a person*	3.57 (1.90-6.71)	1.93 (0.83-4.47)	0.13
	*Unable*	11.62 (6.39-21.10)	1.93 (0.74-5.04)	0.18
**Bathing**	*Independent*	1.00 (Reference)	1.00 (Reference)	-
	*Dependent*	0.13 (0.04-0.44)	1.88 (0.38-9.29)	0.44

^b^N varies between 943 and 980 in bivariate analysis and N = 868 in multiple logistic regression.

^c^The multivariable logistic regression model consists of the 14 covariates in the table.

^d^P-values for the multivariable logistic regression model.

### Constipation, diarrhea and fecal incontinence

The registered nurses reported that 5.9% of the residents were constipated when those treated with laxatives were excluded. Including residents treated with laxatives, the number of residents reported with constipation was 41%. The prevalence of diarrhea or loose stool was 17.3%. Fecal incontinence occurred in nearly half (49%) of the residents with constipation (p = 0.001), but in the multivariable logistic regression model there was no significant association between constipation (including laxative use) and fecal incontinence. Fecal incontinence occurred in 78.0% of the cases with diarrhea (p < 0.001).

### Correlates of fecal incontinence

The variables age, sex, diabetes, Parkinson’s disease, hip problems, depression, and sight reduction were not significantly associated with fecal incontinence in bivariate analysis and therefore not included in the multivariable logistic regression model. Table 3 demonstrates the multivariable regression model consisting of 14 independent variables and with a 50.2% goodness of fit. According to this model, residents with diarrhea or loose stool had nearly 7.5 times higher odds of having fecal incontinence (OR 7.33, CI 4.39-12.24), residents with urinary incontinence had over 2.5 times higher odds of having fecal incontinence (OR 2.77, CI 1.73-4.42) and residents with dementia had over 2 times higher odds of having fecal incontinence (OR 2.17, CI 1.28-3.68) compared to those without the condition. Residents who had been residing in a nursing home between 4–5 years were more than 2.5 times more likely to have fecal incontinence compared to residents who had stayed in a nursing home under a year (OR 2.65, CI 1.20-5.85).

Regarding ADL-functioning, a significant association was found between impaired feeding, dressing, toilet use, mobility and fecal incontinence (Table 3). Deficiency in one of these ADL-functions gave higher odds of having fecal incontinence compared to residents without these ADL-deficiencies. However, residents who needed some help for transfer between bed and chair had less than half the likelihood of having fecal incontinence compared to residents who transferred independently (OR 0.49, CI 0.26-0.91). The other variables in Barthel’s ADL index; grooming, stairs and bathing had no significant association with fecal incontinence in this model.

## Discussion

This nursing home population-based study demonstrates that fecal incontinence is prevalent in nursing homes and that fecal incontinence correlates with a number of different medical conditions. Our study had a large sample size, high response rate and is highly representative of the present older and frailer nursing home population in Norway.

### Prevalence of fecal incontinence

The findings of 42.3% nursing home residents with fecal incontinence at least a few times a month or more often corresponds well with other studies with a prevalence of fecal incontinence between 40-55% [5-10]. A cross sectional study of certified nursing home facilities in USA during 2005 found the prevalence of fecal incontinence more often than once a week to be 43.0% [8]. This may be interpreted as a higher prevalence in US nursing facilities since this is the same prevalence, but they considered weekly rather than monthly fecal incontinence. However, it is difficult to compare results from different nursing home units in different studies, since nursing homes are heterogeneous across the world [27], when resident characteristics and the kind of care given in nursing homes are taken into account. One could anticipate a higher prevalence when the nursing home population is older and frailer than in previous studies, but this does not seem to be the case. Instead the prevalence seems to be stable. Hopefully, this may be due to improved fecal incontinence treatment and care. The prevalence of fecal incontinence among short-term care residents was 17.3%, compared to 44.1% for residents in long-term care. These differences are hardly surprising as this may be an expression of an increasing frailty; residents in short-term care are likely to have a higher level of functioning than residents in long-term care.

There was no significant difference in the prevalence of fecal incontinence between men and women. However, we found a trend towards higher prevalence in women than among men in bivariate analysis. Fecal incontinence has been linked to childbirth and birth injuries, although some studies have indicated a higher prevalence of fecal incontinence in men than in women among nursing home residents [1,6,9]. The prevalence of fecal incontinence did not differ significantly according to age. A systematic review of fecal incontinence in community-dwelling people clearly demonstrated that increasing age was a significant risk factor for fecal incontinence [2]. However, adding the independent variables sex and age to the multiple logistic regression model did not make any difference; either sex or age was significantly associated with fecal incontinence, and they did not change the outcome of the other correlates. This suggests that when residents have reached the stage where they are admitted to a nursing home, age is no longer a key factor for developing fecal incontinence.

### Bowel problems

There are few studies of prevalence of constipation in nursing homes. In an American cross sectional study prevalence is reported as 12.5%, excluding residents using laxatives [28]. It is, however, more common to take laxative use as a marker for constipation and such studies report prevalence from 47% to 56% [29-31]. The prevalence of constipation in our study was 41.0% when laxative use was a marker. Several studies have demonstrated a significant association between constipation or fecal impaction and fecal incontinence [7,10,16,20]. The bivariate analysis of constipation and fecal incontinence showed significant association (OR 1.55, CI 1.20-2.01). However, adjusted for other factors in a logistic regression model, constipation was not significantly correlated to fecal incontinence (Table 3). Constipation and fecal impaction may lead to widening of the ano-rectal angle, a reduction of rectal sensation and an increase in the stool volume needed to trigger the rectal–anal inhibitory reflex [32]. Johansen, however, found hard stool to be a protective factor against fecal incontinence [9]. If feces are hard and cannot be passed, leakage is less likely unless impaction has developed. Use of laxatives in this group is high and has in some studies been regarded as a cause of fecal incontinence [23,33,34]. Brocklehurst [6] found an association between some stimulant laxatives and fecal incontinence. We did not find any significant association between laxative use and fecal incontinence in our study.

The prevalence of diarrhea in nursing homes is even more rarely reported [35]. In our study the prevalence of diarrhea was 17.3%. We do not know the cause of diarrhea, but we consider it to be a major concern in nursing homes and a severe issue for the residents concerned. Diarrhea or loose stool has shown to be a significant predictor of fecal incontinence in nursing home residents [7,20,36]. With or without a weakness in the external anal sphincter, diarrhea or loose stool may lead to fecal incontinence [37]. In many cases the resident has an additional physical and/or cognitive impairment. A sudden “urge” to empty the bowels may therefore explain why 52.7% of our population had a problem to reach the toilet in time to empty their bowels.

### Functional incontinence

Reduced ADL-functioning is an important reason for nursing home admission [38]. This affects the residents’ ability to control leakage of feces. Residents with dementia or cognitive impairment had 2.17 times higher odds of having fecal incontinence than residents who did not have cognitive impairment. To be unable to eat, dress, move or use the toilet independently were each significantly associated with fecal incontinence and were the factors with the highest OR (2.17-7.37). To be unable to move, dress or use the toilet independently are together with the cognitive aspect essential factors in being able to manage one’s bowels independently. It is obvious that these functions correlate to fecal incontinence. Bowel control is related to the ability to cognitively know that you need to go to the toilet, to know where the toilet is, to be able to move to the toilet, to be able to undress and then dress, to have the dexterity to undress and to be able to get help from someone if needed. P-values for the variables dressing and mobility are respectively 0.01 and 0.03 and these results must therefore be interpreted with caution.

Residents with urinary incontinence had 2.77 times higher odds of having fecal incontinence than residents with no urinary incontinence. This may be an expression of the same functional difficulties with maintaining continence. Another explanation may be that this is related to a general pelvic floor problem in the resident. Table 3 demonstrates significant association between length of stay and fecal incontinence, though only in residents that had resided in a nursing home between 4 and 5 years (p = 0.02), but this result must be interpreted with caution. It is however possible that residents lose their continence as a consequence of nursing home residency. A study of incidence of fecal incontinence in nursing home residents showed a cumulative incidence of 20% within the first 10 months of residency [7]. Moving from a familiar home environment to a nursing home can be a life-changing event; a new physical environment, new routines, unfamiliar nursing home staff and a new life-situation may be stressful for a resident who is already in a state of progressing physical and mental decline. This may over time lead to confusion, passiveness, and lack of control over bodily functions. Interestingly, needing some help for transfer from bed to chair was a protective factor for fecal incontinence (Table 3). Possibly, the residents’ bowels are more likely to be well looked after when the resident needs this kind of assistance from staff, while independent residents do less well. This may be an expression of the important role registered nurses have in fecal incontinence care. Fecal incontinence is not only about resident disabilities, but also how well the registered nurses are able to give care that compensate for resident disabilities. However, needing some help in transfer from bed to chair had a p-value of 0.03, which means that this result should be interpreted with caution.

### Fecal incontinence management

The variables associated with fecal incontinence in both the current and previous studies, are to a large extent remediable factors. In order to prevent the development of fecal incontinence and to achieve better management and treatment practices in this respect, a structured multifactorial assessment is required. Bowel patterns and history of bowel symptoms should be a central part of the assessment for all nursing home residents and rectal examination should be performed to evaluate fecal impaction [39]. There was no significant association between constipation and fecal incontinence in our study. Nevertheless, fecal impaction has been associated with overflow incontinence in previous studies [7,40] and should be taken into account when managing fecal incontinence in nursing home residents. Additionally, pelvic examination should be performed, especially when the resident has double incontinence, in order to identify possible prolapse or rectocele [41]. Cognitive and functional assessments are important to evaluate the resident’s ability to access and use the toilet. In this group of frail elderly it may be unrealistic to expect great improvement of fecal incontinence or cure. However, a Norwegian study showed a significant decrease in urine leakage among residents participating in an individualized training program designed to improve ADL and physical capacity. By actively training residents, worsening of urinary incontinence was prevented or reduced [42]. Prompted toileting has been tested for dementia-related fecal incontinence, but the results were not convincing [43]. Residents identified as having constipation with overflow should have an effective bowel clearance [6,40] and the staff should always have a strong focus on prevention of constipation. According to the findings in our study, it seems important that residents with diarrhea and loose stool receive treatment. There are no data regarding the use of Loperamide for the frail elderly, but expert opinion suggests extreme caution and monitoring for impaction when used in this patient group. In the case of C. difficile infection, appropriate preventive measures should be taken [1]. For residents without significant cognitive problems, both biofeedback treatment and pelvic floor retraining has shown to be effective in respectively older incontinent people and older women with urinary incontinence [44,45]. The association between length of stay and fecal incontinence in this population should be explored further. However, as this may be an indication of the quality of care and treatment given, it seems like fecal incontinence lacks the priority and attention that it deserves from health professionals in nursing homes.

Fecal incontinence is associated with discomfort, pain, embarrassment and loss of dignity. For nursing home staff it also implies an extra and unpleasant workload. It is therefore important to prolong and maintain such an important function as continent emptying of the bowels. Although there is a need to establish new reliable knowledge regarding the management of fecal incontinence among nursing home residents, there are few published trials of treatment of fecal incontinence among nursing home residents, and no trials on prevention. The existing studies had small numbers, problematic methodology, and were all non-blinded [1].

### Limitations and strength of the study

It might be considered a weakness that residents in this study did not fill in the questionnaires themselves. However, approximately 80% of nursing home residents suffer from dementia or cognitive impairment and this make nursing home residents a very difficult population in which to do research. Thus, we designed a study where registered nurses filled in the questionnaire on behalf of the residents. If we had included only residents without cognitive impairment, the study would not have been representative of the nursing home population. Family or caregivers might be used as informants, but family members seldom have anything to do with the resident’s bowel habits or bowel problems; this is a natural area for professional caregivers to manage. Nevertheless, it may be questioned as to how well professional caregivers really know their resident concerning these intimate subjects, especially since caregivers have many residents to attend to. On the other hand, it is important to stress that in Norwegian nursing homes, registered nurses are not just doing administrative work tasks, they also participate in resident activities such as personal hygiene and toileting. It is therefore expected that they are closely familiar with the residents’ bowel habits.

We did not distinguish between constipation and fecal impaction. To identify fecal impaction we would have needed to do a rectal examination on all residents. This was not feasible. It is also important to highlight that constipation or rectal impaction may be a source of error regarding fecal incontinence. Residents do not have leakage at the time when they are constipated, except if they have “spurrous diarrhea” relative to impaction. This again is a possible source of error regarding diarrhea: spurrous diarrhea may be misinterpreted as diarrhea, when it is really caused by constipation. However, this is not possible to distinguish here.

In our study we used Barthel’s ADL index and frequency labeling from the severity index [25], which are validated instruments. However, other questions used in our survey are not validated due to a lack of Norwegian questionnaires available. In our questionnaire, we have included questions representing cornerstone issues in measuring fecal incontinence in nursing homes, and they are clearly formulated and easy to understand for the registered nurse. The questionnaire was also pilot tested in one nursing home unit. Some changes were made according to feedback from the pilot test, but the general impression from the pilot test was that the questions were highly understandable and not subject to discussions. Nevertheless, there may still be discrepancies regarding how registered nurses interpreted the questions. Other formal information about the questionnaire’s reliability and validity do not exist. Initially we wanted to include questions about diet, fluids and parity in the questionnaire, but decided that it would be difficult for the registered nurse in the nursing homes to gather reliable information about these factors. Instead we prioritized questions that could be collected easily by the registered nurse.

In this study we wanted to explore further the strength of association between correlates identified in previous studies and fecal incontinence. We included 14 independent variables in the multivariable regression model. We are aware of the problem of increased rates of false positive testing when carrying out multiple testing, but conventional adjustments for multiple comparisons are considered too conservative at the expense of false negative findings [46]. Thus, we did no further adjustments in our analysis. However, owing to multiple testing P-values between 0.01 and 0.05 are interpreted with caution.

The strength of this study is the high response rate; 90.3% within the nursing home population and 100% in the participating nursing homes. The nursing home that chose not to participate in the study (80 residents) is highly representative compared with the included cases, both in the distribution of long-term care/short-term care, but also in resident characteristics. The unit that declined participation in another nursing home had 30 residents and many of them were short-term care residents.

It is difficult to make international comparisons of nursing homes because countries across the world have different standards of care, resources and attitudes in nursing homes. Historically, nursing homes have different backgrounds and different organizational and financial systems [38]. Nevertheless, nursing home residents are institutionalized and frail elderly residents, they are among the oldest in our societies, and they are all in need of professional care. In this regard, this study from Norway is representative of nursing homes in other countries, especially in the western world. Hence, our results can provide knowledge about prevalence and correlates of fecal incontinence, which is of great importance in the planning of resident care.

## Conclusions

This study has provided a thorough investigation of fecal incontinence related to sex, age, length and type of stay in nursing home, bowel symptoms, medical conditions and ADL functioning. We found that prevalence of fecal incontinence among nursing home residents was 42.3% and this study confirms that fecal incontinence is a common condition among nursing home residents. Despite the increase in age and frailty in nursing home residents over the past decades, we found no major change in prevalence of fecal incontinence in this population compared to previous studies. We found a significant association between diarrhea, nursing home stay between 4–5 years, urinary incontinence, dementia, reduction in ADL-functioning and fecal incontinence. In a resident group with likely deteriorating health and obvious needs, there still remain unmet needs to prolong and maintain such an important function as continent emptying of the bowels.

Overall, the findings of this study have the potential to contribute directly to the improvement of clinical practice in nursing homes. This is because fecal incontinence is a highly prevalent condition in nursing homes worldwide and therefore new knowledge is of interest for all multidisciplinary staff working with elderly residents in nursing homes. For caregivers, fecal incontinence may be seen as a normal condition linked to the aging process and frailty, but being able to maintain bowel continence is a question of dignity for the resident in the late stages of life. This is a complex phenomenon which requires in depth knowledge and understanding regarding causes and consequences for the resident. Making an individual multifactorial assessment of the residents is necessary regarding bowel assessment and assessment of mental and physical capacity. Consequently, individual treatment and care planning are needed to meet this multifaceted problem. More research on how to promote and prolong bowel continence among frail elderly residents is required.

## Abbreviations

ADL: Activities of daily living; CI: Confidence interval; OR: Odds ratio; SD: Standard deviation.

## Competing interests

The authors declare that they have no competing interests.

## Authors’ contributions

SS participated in the design of the study, the data collection, performed the statistical analysis, interpretation of results and drafted the manuscript. AS participated in the design of the study, performed the statistical analysis and participated in the interpretation of the results. AGV, SM and CN participated in the design of the study and in the interpretation of the results. All authors read and approved the final manuscript.

## Pre-publication history

The pre-publication history for this paper can be accessed here:

http://www.biomedcentral.com/1471-2318/13/87/prepub
